# Endothelial dysfunction and persistent inflammation in severe post-COVID-19 patients: implications for gas exchange

**DOI:** 10.1186/s12916-024-03461-5

**Published:** 2024-06-13

**Authors:** Enrique Alfaro, Elena Díaz-García, Sara García-Tovar, Raúl Galera, Raquel Casitas, María Torres-Vargas, Cristina López-Fernández, José M. Añón, Francisco García-Río, Carolina Cubillos-Zapata

**Affiliations:** 1 Respiratory Diseases Group, Respiratory Service, La Paz University Hospital, IdiPAZ, Paseo de La Castellana 261, 28046 Madrid, Spain; 2 Biomedical Research Networking Centre On Respiratory Diseases (CIBERES), Madrid, Spain; 3 Department of Intensive Medicine, La Paz University Hospital, Madrid, Spain; 4 Faculty of Medicine, Autonomous University of Madrid, Madrid, Spain

**Keywords:** Acute respiratory distress syndrome, Post-COVID-19 syndrome, Endothelial dysfunction, ICAM-1, Inflammation

## Abstract

**Background:**

Understanding the enduring respiratory consequences of severe COVID-19 is crucial for comprehensive patient care. This study aims to evaluate the impact of post-COVID conditions on respiratory sequelae of severe acute respiratory distress syndrome (ARDS).

**Methods:**

We examined 88 survivors of COVID-19-associated severe ARDS six months post-intensive care unit (ICU) discharge. Assessments included clinical and functional evaluation as well as plasma biomarkers of endothelial dysfunction, inflammation, and viral response. Additionally, an in vitro model using human umbilical vein endothelial cells (HUVECs) explored the direct impact of post-COVID plasma on endothelial function.

**Results:**

Post-COVID patients with impaired gas exchange demonstrated persistent endothelial inflammation marked by elevated ICAM-1, IL-8, CCL-2, and ET-1 plasma levels. Concurrently, systemic inflammation, evidenced by NLRP3 overexpression and elevated levels of IL-6, sCD40-L, and C-reactive protein, was associated with endothelial dysfunction biomarkers and increased in post-COVID patients with impaired gas exchange. T-cell activation, reflected in CD69 expression, and persistently elevated levels of interferon-β (IFN-β) further contributed to sustained inflammation. The in vitro model confirmed that patient plasma, with altered levels of sCD40-L and IFN-β proteins, has the capacity to alter endothelial function.

**Conclusions:**

Six months post-ICU discharge, survivors of COVID-19-associated ARDS exhibited sustained elevation in endothelial dysfunction biomarkers, correlating with the severity of impaired gas exchange. NLRP3 inflammasome activity and persistent T-cell activation indicate on going inflammation contributing to persistent endothelial dysfunction, potentially intensified by sustained viral immune response.

**Supplementary Information:**

The online version contains supplementary material available at 10.1186/s12916-024-03461-5.

## Background

Acute COVID-19, resulting from SARS-CoV-2 infection, exhibits a broad clinical spectrum, from asymptomatic cases to fatalities. Acute respiratory distress syndrome (ARDS) significantly contributes to COVID-19-related mortality [1], causing respiratory failure and multi-organ dysfunction [2]. Besides acute lethality, ARDS profoundly affects survivors [3]. In fact, the process of alveolar-capillary membrane permeabilization and repair often leaves some ARDS survivors with persistent alterations in gas exchange, which exacerbates symptoms and decreases quality of life [4, 5].

In COVID-19 ARDS survivors, medium-term clinical and functional sequelae may coincide with the persistence of symptoms beyond the acute phase, originating from a set of physical, cognitive, mental and respiratory alterations, collectively referred to as long-term or post-COVID-19 condition [6–9]. Increasing evidence suggests the emergence of this new complex systemic condition, affecting a proportion of patients ranging from 10 to 30% [9]. The severity of COVID-19 during the acute phase, particularly with ARDS, heightens the risk of persistence of symptoms and lung function impairment [10].

Primary pathophysiological mechanisms of acute COVID-19 may contribute to post-acute COVID-19 [2], involving virus-specific changes and inflammatory damage. Virus-dependent mechanisms, including severe acute respiratory syndrome coronavirus-2 (SARS-CoV-2)-infected endothelial cells [11], and virus-independent mechanisms, such as immune damage and inflammation, collectively contribute to the breakdown of the endothelial-epithelial barrier [12]. These processes induce cellular damage, innate immune response, inflammatory cytokine production, and a procoagulant state by SARS-CoV-2, all impairing gas exchange [13].

We hypothesize that persistent systemic inflammation in post-COVID-19 patients may contribute to endothelial dysfunction, initiating endothelial harm and leading to clinical and functional impairment. Consequently, we assessed endothelial and systemic inflammatory biomarkers six months post-severe ARDS secondary to COVID-19. This evaluation, coupled with an examination within an endothelial cell model, aims to elucidate potential underlying mechanisms.

## Methods

### Study subjects

We enrolled consecutive participants, aged 18 or older, who survived severe ARDS associated with COVID-19, meeting Berlin criteria and requiring invasive mechanical ventilation for at least 7 days. SARS-CoV-2 infection was confirmed by positive reverse-transcriptase polymerase chain reaction on nasal swab or tracheal aspirate at the time of ARDS. Detailed selection criteria are in the Additional file 1 [14–29]. Written consent was obtained from all participants, and the study received approval from the institutional Ethics Committee (PI-4189).

For ARDS survivors, demographic, clinical, and ICU management data were recorded. Six months post-ICU discharge, anthropometric parameters, smoking status, comorbidities, and current treatment were documented. Respiratory symptoms were assessed using the European Community for Coal and Steel Questionnaire and the modified Medical Research Council dyspnoea scale [16, 17]. Health-related quality of life was measured with the Spanish version of the Medical Outcomes Study 12-Item Short-Form Health Survey (SF-12) [18]. Spirometry and diffusing capacity of the lungs for carbon monoxide (DLCO) measurements were conducted using MasterScreen (Viasys, CareFusion, Germany) according to current standardization, with Global Lung Initiative equations as reference values and interpretations following European Respiratory Society (ERS)/American Thoracic Society (ATS) strategies [23]. Detailed information on all clinical and functional measurements is available in the Additional file 1.

### Peripheral blood mononuclear cells isolation and monocytes culture

18 mL of blood were obtained from COVID-19-associated ARDS survivors by venipuncture into EDTA tubes. Blood was layered on top of 10 mL Ficoll-Paque Plus (Amersham Biosciences, Amersham, UK) and centrifuged at 1500 rpm for 20 min at 24 °C. Plasma was removed from the upper layer; peripheral blood mononuclear cells (PBMCs) were removed from the interphase and washed in phosphate buffered saline for later culture or to allow flow cytometry quantifications.

For enriched monocytes culture, PBMCs were placed on M6 plates (5 × 10^5^ monocytes/well) in 1 mL RPMI 1640 medium (Ref: 52,400–025, Thermofisher Scientific, MA, USA) supplemented with 1% penicillin/streptomycin. After 45 min medium including non-adherent cells is withdrawn leaving enriched monocytes culture in the plate. 0.5 mL of medium supplemented with 10% fetal bovine serum is added to the cells and cultured for 16 h.

### Human umbilical vein endothelial cells culture

Human umbilical vein endothelial cells (HUVECs) were purchased from Innoprot (Ref: P10961; Bizkaia, Spain) and were cultured in fibronectin-coated plates (2 µg/cm^2^) with endothelial cell medium from Innoprot (Ref: P60104) at 37ºC and 5% CO_2_. Subculture was performed following manufacturer instructions. Briefly, cells at 90% confluency were rinsed with Dulbecco’s PBS (DPBS) and trypsin 0.025% solution was added and incubated at 37ºC for 2–5 min until cells completely round up. Detached cells in trypsin solution were recovered and trypsin was neutralized using fetal bovine serum and centrifuged for 7 min at 1200 rpm. Lastly, cells were counted and plated accordingly in fibronectin-coated plates.

### Flow cytometry

Cells were harvested from culture and labelled with specific antibodies. Permeabilization and staining protocol details are included in the Additional file 1. The cells were acquired using a BD FACS-Calibur flow cytometer (Becton–Dickinson Biosciences, RRID:SCR_000401) and the collected data were analysed using FlowJo v10 (Becton–Dickinson Biosciences).

### Plasma soluble markers

Plasma soluble markers were quantified by ELISA assays following manufacturer’s instructions. Detailed information about ELISA kits employed in this study can be found in Additional file 1: Table S1.

Plasma cytokines were measured using BD Human Inflammatory Cytokine cytometric bead array (CBA) kit (Ref: 551,811, Becton–Dickinson Biosciences), acquired by BD FACS-Calibur flow cytometer and analysed by FCAP Array software (Becton–Dickinson Biosciences).

### mRNA isolation and qPCR analysis

RNA was extracted from PBMCs or HUVECs, retrotranscribed and cDNA was quantified by real-time qPCR using specific primers (Additional file 1: Table S2). Results were normalized to housekeeping gene 18S. Detailed protocol and reagents can be found in the Additional file 1.

### Statistical analysis

Assuming a mean deviation of 98 ng/ml for the plasma concentration of intercellular adhesion molecule 1 (ICAM-1) (data from a preliminary pilot study), to detect a difference of at least 55 ng/ml with an alpha error of 0.05, a beta error of 0.20, and a dropout rate of 0.10, a minimum of 27 patients per group would be required. Considering that up to 31% of post-COVID-19 ARDS survivors may exhibit reduced DLCO, it would be necessary to include 88 patients in both groups to achieve the estimated sample size.

Categorical variables are presented as numbers with percentages, and continuous variables as mean with standard error of the mean (SEM) or median (95% confidence interval), according to their distribution. Comparisons between subgroups were performed using the t Student, Mann–Whitney or chi-squared tests. For more than two groups, mean differences were evaluated using one-way ANOVA with Tukey’s test multiple comparison or two-way ANOVA with multiple comparison Sidak’s test. The relationship between variables was assessed with the Pearson and Spearman correlation analysis. Data were analysed with the GraphPad Prism v.8 and SPSS 25.0 software, considering *P*-values < 0.05 statistically significant for all tests. Partial least squares-discriminant analysis (PLS-DA) model was calculated in R-Studio software using “caret” package. Variable importance projection scores were calculated with “vip” and “dplyr” packages and plotted with “ggplot2” and “ggalt” packages.

## Results

### Characteristics of the study subjects

Detailed characteristics of ARDS survivors are presented in Table 1 and Additional file 1: Table S3 and Table S4. Notably, among the 88 patients assessed, 29 (33%) exhibited reduced DLCO six months post-ICU discharge. Additionally, 66 patients (75%) reported respiratory symptoms, with 36 cases (41%) indicating a dyspnea level of 2 or higher on the mMRC scale persisting since the time of hospital discharge. Furthermore, patients with decreased DLCO also showed lower exercise tolerance as well as a greater degree of gas exchange impairment, increased dead space effect, lower oxyhaemoglobin saturation, and greater exertional dyspnoea (Additional File 1: Table S5). Thus, DLCO clearly distinguishes individuals with poorer gas exchange and oxygenation during exercise.

**Table 1:** General characteristics of the study subjects

**Characteristic**	**Overall group** (*n* = 88)	**Subjects with normal DLCO** (*n* = 59)	**Subjects with decreased DLCO** (*n* = 29)	***p*-Value**
Males, n (%)	61 (69.3)	41 (69.5)	20 (69.0)	0.573
Age, years	59 ± 10	59 ± 11	59 ± 8	0.873
Body mass index, Kg/m^2^	29.9 ± 4.9	30.5 ± 4.8	28.6 ± 5.0	0.104
Fat mass index, Kg/m^2^	10.4 ± 4.5	10.7 ± 4.6	9.7 ± 4.1	0.294
Current or former smokers, n (%)	27 (30.6)	19 (32.2)	8 (27.6)	0.738
Main comorbidities, n (%)				
Hypertension	53 (60.2)	36 (61.0)	17 (58.6)	0.504
Dyslipidemia	41 (46.6)	29 (49.2)	12 (41.4)	0.324
Obesity	35 (39.8)	23 (39.0)	12 (41.4)	0.504
Diabetes	14 (15.9)	11 (18.6)	3 (10.3)	0.250
Hypothyroidism	15 (17.0)	11 (18.6)	4 (13.8)	0.404
Cardiovascular diseases	12 (13.6)	6 (10.2)	6 (20.7)	0.154
Respiratory diseases	7 (8.0)	5 (8.5)	2 (6.9)	0.580
Number of prior comorbidities	1 (0–1)	1 (0–1)	1 (0–1)	0.495
ICU admission parameters				
APACHE-II	16 ± 5	16 ± 5	14 ± 4	0.175
PaO_2_/FiO_2_	118 ± 52	116 ± 50	121 ± 55	0.643
Lymphocytes, × 10^3^/µl	800 (450–840)	610 (430–810)	820 (625–845)	0.135
D-dimer, ng/ml	2106 (1386–3549)	2232 (1386–3549)	1771 (929–38,928)	0.787
C-reactive protein, mg/L	268 (211–283)	277 (268–283)	179 (81–318)	0.076
IL-6, pg/ml	143 (46–544)	143 (113–153)	274 (4–772)	0.635
Invasive mechanical ventilation duration, days	24 (12–90)	24 (12–42)	67 (16–117)	0.055
Severity of ADRS (Berlin definition)				0.841
Moderate	20 (22.7)	13 (18.6)	7 (24.1)	
Severe	68 (77.3)	46 (78.0)	22 (75.9)	
Respiratory parameters on intubation				
Plateau pressure, cmH_2_O	26 ± 5	25 ± 5	27 ± 5	0.105
Peak inspiratory pressure, cmH_2_O	32 ± 5	31 ± 6	33 ± 4	0.424
Positive end-expiratory pressure, cmH_2_O	11 ± 3	11 ± 3	12 ± 4	0.136
Driving pressure, cmH_2_O	12 ± 1	11 ± 1	13 ± 2	0.196
Static compliance, ml/cmH_2_O	38 ± 13	38 ± 11	38 ± 15	0.980
Prone position, n (%)	71 (80.7)	47 (79.7)	24 (82.8)	0.485
Tracheostomy, n (%)	44 (50.0)	29 (49.2)	15 (51.7)	0.500
Extracorporeal membrane oxygenation, n (%)	4 (4.5)	2 (3.4)	2 (6.9)	0.401
Complications during ICU stay, n (%)				
Nosocomial infection	48 (54.5)	34 (57.6)	14 (48.3)	0.274
Pleural effusion	6 (6.8)	4 (6.8)	2 (6.9)	0.649
Pulmonary thromboembolism	25 (28.4)	19 (32.2)	6 (20.7)	0.192
ICU-acquired weakness	37 (42.0)	25 (42.4)	12 (41.4)	0.558
Hyperactive delirium	28 (31.8)	19 (32.2)	9 (31.0)	0.557
Reintubation	8 (9.1)	5 (8.5)	3 (10.3)	0.527
ICU readmission	4 (4.5)	3 (5.1)	1 (3.4)	0.599
Weight loss in ICU, Kg	12 (5–20)	17 (5–20)	11 (6–42)	0.791
Home oxygen therapy at discharge, n (%)	11 (12.5)	4 (6.8)	7 (24.1)	0.027
ICU length of stay, days	24 (12–46)	25 (10–56)	23 (12–30)	0.975
Hospital length of stay, days	43 (25–81)	43 (25–88)	40 (25–72)	0.849

Values are mean ± standard deviation, median (interquartile range) or number (percentage) according to their type and distribution. *Abbreviations*: *ICU* Intensive Care Unit, *APACHE* Acute Physiology, Age, and Chronic Health Evaluation, *DmCO* Diffusing capacity of the membrane, *PaO*_*2*_*/FiO*_*2*_ Ratio of arterial oxygen partial pressure to fractional inspired oxygen, *IL-6* Interleukin 6, *VA* Alveolar volume

### Persistence of endothelial dysfunction biomarkers in COVID-19 ARDS survivors with impaired gas exchange

**Fig. 1:**
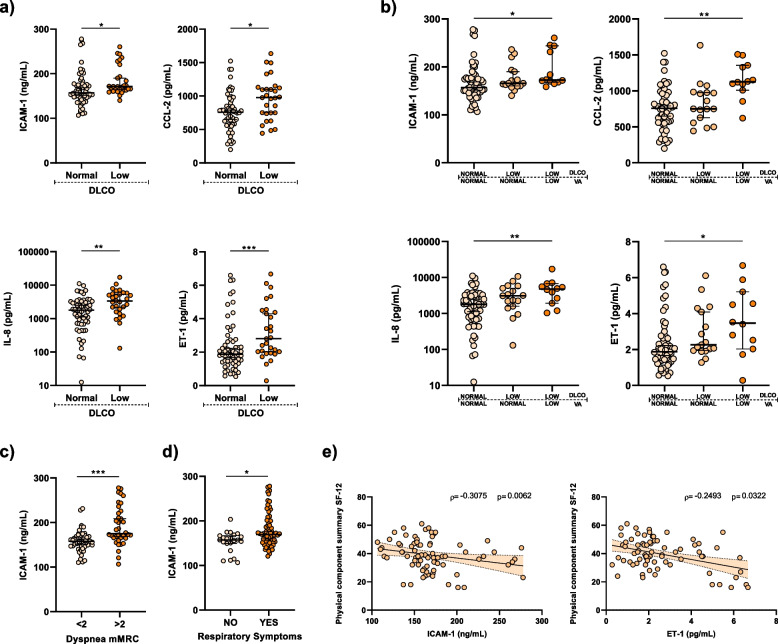
Elevated levels of endothelial dysfunction biomarkers in post-COVID-19 patients with impaired gas exchange. **a** Plasma levels of ICAM-1 (left-upper panel), IL-8 (left-lower panel), CCL-2 (right-upper panel), and ET-1 (right-lower panel) were quantified using ELISA for post-COVID-19 patients with normal (*n* = 59) and low DLCO (*n* = 29). **b** Post-COVID-19 plasma levels of ICAM-1 (left-upper panel), IL-8 (left-lower panel), CCL-2 (right-upper panel), and ET-1 (right-lower panel) based on DLCO and alveolar volume (VA). **c** Comparison of ICAM-1 plasma levels in post-COVID-19 patients based on dyspnoea mMRC scale (< 2 *N* = 52; > 2 *N* = 36). **d** Comparison of ICAM-1 plasma levels in post-COVID-19 patients without respiratory symptoms (*N* = 22) and patients with persistent respiratory symptoms (*N* = 66). **e** Spearman correlation of ICAM-1 (left panel) and ET-1 (right panel) plasma levels with the physical component summary of the SF-12 questionnaire. Data are represented as median ± 95% confidence interval. Group differences were analysed by Mann–Whitney U test or by one-way ANOVA with Tukey’s multiple comparison test. Spearman correlation coefficients (ρ) and *p*-values (p) are indicated. *P* values are denoted as follows: *p < 0.05; **p < 0.01; ***p < 0.001

At 6 months post-ICU discharge, ARDS survivors (PCOV or post-COVID-19) with impaired gas exchange, characterized by reduced DLCO exhibited elevated plasma levels of intercellular adhesion molecule-1 (ICAM-1), interleukin-8 (IL-8), chemokine (C–C motif) ligand 2 (CCL-2) and endothelin-1 (ET-1) compared PCOV patients with normal DLCO (Fig. 1a).

When comparing post-COVID-19 patients with normal or low DLCO, stratification based on alveolar volume (VA) revealed differences in ICAM-1, IL-8, CCL-2, and ET-1 levels exclusively in the subgroup with low DLCO and reduced VA (Fig. 1b). Notably, in the absence of high DLCO/VA, this subgroup reflects a loss of alveolar capillary structure and lung volume. In accordance, markers of endothelial dysfunction (ICAM-1 and ET-1) inversely correlated with the percentage of predicted DLCO (Additional file 1: Fig. S1a) and percentage of predicted DLCO/VA (Additional file 1: Fig. S1b). PCOV patients stratified by mMRC dyspnoea level ≥ 2 or by the presence of respiratory symptoms presented differences in plasma concentrations of ICAM-1 (Fig. 1c-d). Finally, an inversely proportional relationship was identified between ICAM-1 and ET-1 plasma levels and the quality of life of PCOV patients, as assessed through the physical component summary of the SF-12 (Fig. 1e).

### Evidence of persistent systemic inflammation in post-COVID-19 patients with impaired gas exchange

**Fig. 2:**
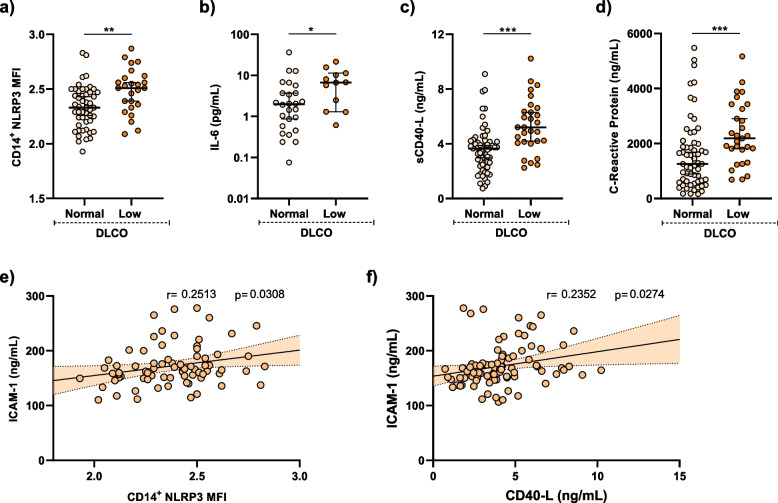
Persistence of systemic inflammation in post-COVID-19 patients with gas exchange impairment. **a** Mean fluorescent intensity (MFI) of NLRP3 in CD14^+^ monocytes from post-COVID-19 patients with normal (*n* = 49) and low (*n* = 25) DLCO. **b** Plasma concentration of IL-6 in post-COVID-19 patients with normal (*n* = 25) and low (*n* = 12) DLCO. **c** Plasma concentration of sCD40-L in post-COVID-19 patients with normal (***n*** = 59) and low (*n* = 29) DLCO. **d** Plasma concentration of C-reactive protein in post-COVID-19 patients with normal (*n* = 59) and low (*n* = 29) DLCO. **e** Linear regression of MFI of NLRP3 in CD14.^+^ monocytes from post-COVID-19 patients and ICAM-1 plasma protein concentration (*n* = 74). F) Linear regression of CD40-L plasma concentration and ICAM-1 plasma protein concentration (*n* = 88). Data are represented as median ± 95% confidence interval. Group differences were analysed by Mann–Whitney U test. Pearson’s correlation coefficients (r) and *p*-values (p) are presented. *P* values are denoted as follows *p < 0.05; **p < 0.01; ***p < 0.001

To evaluate the persistence of systemic inflammation six months post-ICU discharge, we investigated nucleotide-binding oligomerization domain-like receptor 3 (NLRP3) inflammasome expression in circulating monocytes of PCOV patients. Elevated NLRP3 levels were observed in patients with reduced DLCO compared to those with normal DLCO (Fig. 2a). Moreover, the inflammatory cytokine interleukin-6 (IL-6) was increased in the plasma of PCOV patients with low DLCO compared to those with normal DLCO (Fig. 2b). Furthermore, interleukin-1β (IL-1β) and tumor necrosis factor-α (TNF-α) concentrations were similar in monocytes from PCOV patients with low and normal DLCO (Additional file 1: Fig. S2). This suggests the persistent elevation of inflammasome activity in post-COVID-19 patients stratified by gas exchange impairment.

We next examined the plasma concentration of cluster differentiation (CD) 40-ligand (CD40-L), as it can be released to the circulation by platelets and exert strong proinflammatory response of the endothelium, increasing its production of adhesion molecules [30, 31]. We observed an increased concentration of CD40-L in plasma from patients with reduced DLCO (Fig. 2c). Concomitantly, we observed in plasma from patients with low DLCO an increased expression of C-reactive protein, which may reflect the persistence of systemic inflammation in these patients (Fig. 2d). In line, markers of inflammation appeared to be linked to endothelial dysfunction, as indicated by the direct association between plasma ICAM-1 and NLRP3 expression in monocytes and CD40-L concentration in plasma six months after ICU discharge (Fig. 2e-f).

### Persistent T cell inflammation and elevated plasma levels of IFN-β in post-COVID-19 with impaired gas exchange

**Fig. 3:**
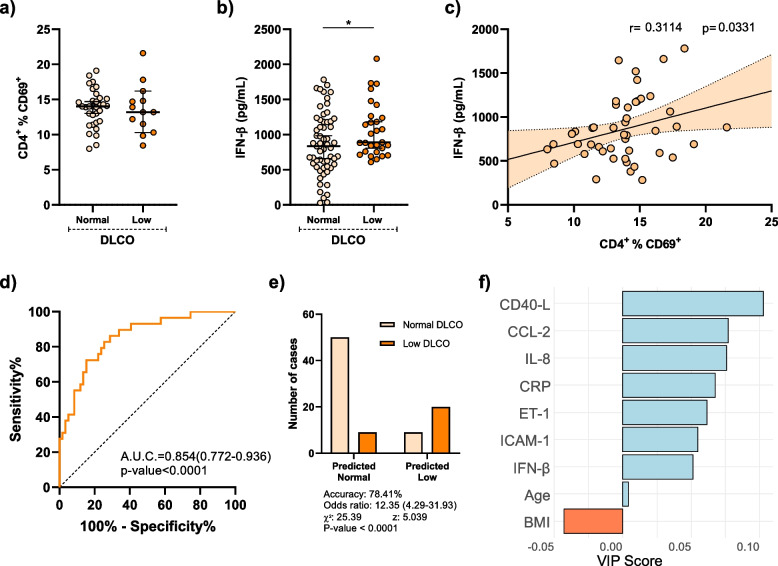
Markers of immune activation, inflammatory response and endothelial dysfunction related to impaired gas exchange. **a** Percentage of CD4^+^ lymphocytes expressing CD69 in post-COVID-19 patients with normal (*n* = 33) and low (*n* = 14) DLCO. **b** IFN-β concentration in plasma from post-COVID-19 patients with normal (*n* = 59) and low (*n* = 29) DLCO. **c** Linear regression of the percentage of activated CD4^+^CD69^+^ lymphocytes and plasma IFN-β concentration in post-COVID-19 patients (*n* = 47). *P* values are denoted as *p < 0.05.Data are represented as median ± 95% confidence interval. Group differences were analysed by Mann–Whitney U test. Pearson’s correlation coefficients (r) and *p*-values (p) are shown. **d**-**f** Partial least square-discriminant analysis (PLS-DA) was performed to integrate values of markers of immune activation, inflammatory response and endothelial dysfunction; body mass index (BMI); and age into a unique score to discriminate patients according to DLCO status. **d** Receiver Operating Characteristic (ROC) curve of PLS-DA score to discriminate patients with normal or reduced DLCO. Area under the curve (A.U.C.) and *p*-value are shown. **e** Contingency plot illustrates the number of patients with normal or low DLCO in the groups predicted by PLS-DA score. Accuracy, odds ratio, chi square, z-value and *p*-value are shown. **f** Variable importance projection (VIP) scores of the different factors implicated in PLS-DA model, which rank factors according to the importance for the model and their association to reduced DLCO (blue: positive relation; orange: negative relation). ROC curve was analyzed by Wilson/Brown test. Contingency table was analyzed by chi-squared test

To elucidate the potential role of persistent T cell inflammation in post-COVID-19 patients, we examined CD4^+^ T cell activation through CD69 expression. Our analysis revealed, six months after ICU discharge, PCOV patients did not show significant difference between normal and reduced DLCO (Fig. 3a). Notably, the presence of activated T cells alongside inflammation led us to explore plasma levels of IFN-β. IFN-β plays a role in modulating the innate and adaptive immune response related to viral defence and has the capability to activate T-lymphocytes. Our observations indicated that, six months post-ICU discharge, post-COVID-19 patients with reduced DLCO maintained significantly higher plasma levels of IFN-β than patients with normal DLCO (Fig. 3b). Moreover, a correlation was identified in post-COVID-19 patients between IFN-β plasma levels and CD69 expression on CD4^+^ T lymphocytes (Fig. 3c).

### Markers of inflammatory response, endothelial dysfunction and immune activation discriminate patients with impaired gas exchange

Based on the multiple findings of the study and the diversity of markers of endothelial dysfunction, systemic inflammation, and immune cells activation we performed a partial least squared-discriminant analysis (PLS-DA) to integrate all these factors into a unified score to discriminate patients with gas exchange impairment. PLS-DA model scores were represented in a receiver operating characteristics (ROC) curve which presented an area under the curve (AUC) of 0.854 (0.772–0.963) with a *p* value < 0.0001 (Fig. 3d). The best fitted cut-off value presented an accuracy of 78.41% to discriminate patients with reduced DLCO (Fig. 3e). Variable importance projection (VIP) scores, which rank the importance to the model of the input values, revealed CD40-L as the most important variable followed by chemoattractant proteins IL-8 and CCL-2 (Fig. 3f).

### Endothelial dysfunction using an in vitro model with plasma from post-COVID-19 patients

To unravel the influence of systemic inflammation and IFN-β on endothelial function, an in vitro study utilizing conventional endothelial model cells (HUVECs) was conducted. The objective was to investigate the impact of CD40-L and IFN-β, both present in plasma from post-COVID-19 patients, on endothelial function. Primary assessments included ICAM-1 expression, caspase-1 functionality, and ET-1 expression in HUVEC cells. Additionally, mRNA expression levels of inflammatory cytokines, NLRP3, nuclear factor-κB (NF-κB), and the transcription factor interferon-inducible protein 16 (IFI-16) were examined as complementary measures.

Dysfunction of HUVECs induced by IFN-β and CD40-L recombinant proteins resulted in increased ICAM-1 expression, as assessed via flow cytometry (Additional file 1: Fig. S3a). The presence of IFN-β and CD40-L also led to elevated active caspase-1, indicating NLRP3 inflammasome activation (Additional file 1: Fig. S3b). Similar outcomes were observed with ET-1 expression (Additional file 1: Fig. S3c).

**Fig. 4:**
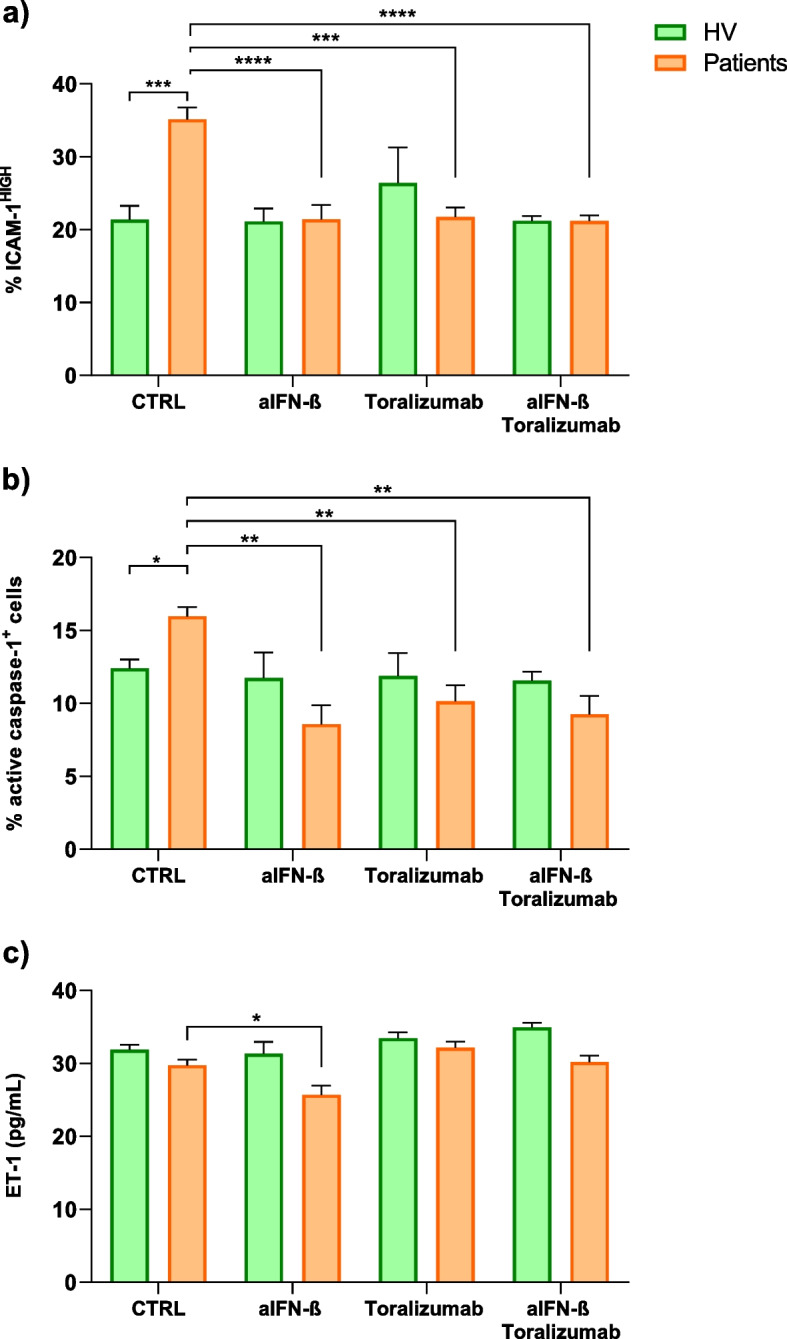
In vitro model of vascular endothelium and soluble proteins from patient’s plasma. HUVECs were cultured for 24 h in the presence of 10% plasma from healthy volunteers (HV, green, *n* = 4) or post-COVID-19 patients (PCOV, orange, *n* = 5). Plasmas were added untreated (CTRL) or previously treated for 16 h with anti-IFN-β antibody (α-IFN-β, 8 μg/mL), anti-CD40-L antibody (Toralizumab, 8 μg/mL), or both. **a** Percentage of HUVECs with high expression of membrane ICAM-1 measured by flow cytometry. **b** Percentage of HUVECs positive for active caspase-1 measured by flow cytometry. **c** Endothelin-1 concentration in supernatants of the cell culture. Bars represent mean ± standard error of the mean (SEM). Mean differences were analysed by two-way ANOVA, and multiple comparisons were performed using Sidak’s test. *p* values are indicated as * *p* < 0.05, ** *p* < 0.01, *** *p* < 0.001, **** *p* < 0.0001

Subsequently, the impact of soluble proteins circulating in plasma on endothelial function was assessed. Patient plasma augmented ICAM-1 expression in HUVECs (Fig. 4a) and increased the percentage of active caspase-1 cells (Fig. 4b) compared to plasma from healthy volunteers. Importantly, blockade of IFN-β and CD40-L attenuated the effect of patient plasma on HUVECs, while it did not impact plasma stimulation from healthy volunteers (Fig. 4a-b). Notably, inhibiting IFN-β resulted in a reduction of endothelin-1 release in the cell culture treated with post-COVID plasma (Fig. 4c). Furthermore, an increase in mRNA expression of ICAM-1, inflammatory cytokines, NLRP3, caspase-1, NF-κB, and IFI-16 was noted, indicating inflammation activity in endothelial cells (Additional file 1: Fig. S4). In summary, plasma from patients, with altered levels of CD40-L and IFN-β proteins, demonstrated the capacity to disrupt endothelial function and activate cell death via caspase-1 activity.

## Discussion

**Fig. 5:**
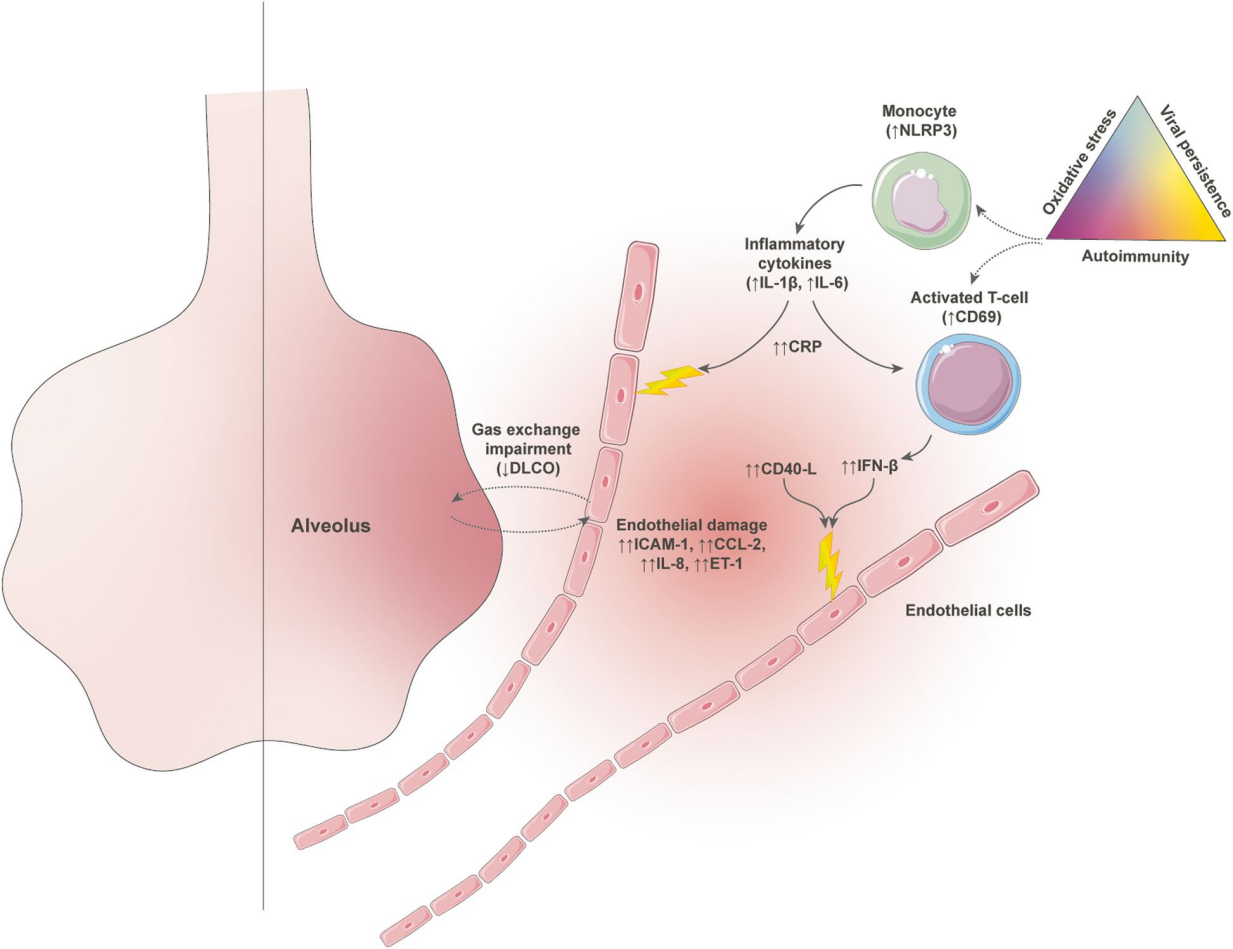
Summary of soluble protein factors in plasma from COVID-19 patients experiencing distress six months post-infection. In post-COVID-19 patients with reduced gas transfer capacity, a noteworthy increase in inflammatory proteins associated with endothelial inflammation is observed. This elevation occurs concomitantly with innate and viral immune responses, inducing heightened cellular stress and consequent escalation of endothelin-1 (ET-1) levels, ultimately resulting in endothelial dysfunction. Specifically, individuals with low DLCO exhibit elevated levels of ICAM-1, IL-8, and CCL-2 proteins, alongside inflammatory proteins (C-Reactive protein (CRP), IL-6 and IL-1β), collectively contributing to cellular stress and activating ET-1 production. Moreover, increased levels of sCD40-L, coupled with IFN-β, in patients with DLCO, impact the endothelial cell model by augmenting ICAM-1 expression and initiating cell death through caspase-1 activity. Additionally, IFN-β influences ET-1 levels. These findings collectively deepen our understanding of inflammation, whether instigated by intercellular cell adhesion or viral inflammation, culminating in endothelial dysfunction mediated by ET-1. This disruption ultimately impedes gas transfer capabilities, underscoring the heightened risk of low lung gas transfer dysfunction in post-COVID patients

In this study, we observed elevated levels of biomarkers associated with endothelial dysfunction in survivors of severe COVID-19-associated ARDS at 6 months post-ICU discharge. Notably, the disparity is more pronounced in post-COVID-19 patients experiencing gas exchange impairment, particularly in those with low DLCO and reduced VA, indicative of alveolar capillary structure loss. Intriguingly, these alterations coexist with a concurrent increase in systemic inflammation biomarkers and serum IFN-β levels, suggesting that persistent systemic inflammation and/or the immune response to the virus may contribute to clinical and functional impairment through their impact on endothelial dysfunction, six months post-acute resolution (Fig. 5). Indeed, our in vitro assays confirm the contributory role of both factors in inducing endothelial dysfunction.

Elevated levels of IL-8, VCAM-1, and ICAM-1 serve as sensitive indicators of endothelial cell alterations induced by nitric oxide imbalance, reactive oxygen species, or thrombotic and inflammatory mediators, leading to disruptions in haemostasis and hemodynamics [32, 33]. Specifically, the expression of ICAM-1 on endothelial cell surfaces or soluble ICAM-1 in plasma is recognized as a biomarker of endothelial cell activation [34]. Additionally, ET-1 release by endothelial cells is triggered by various stimuli, such as angiotensin II, cytokines, free radicals, and reactive oxidative species [35, 36]. The persistence of higher levels of these biomarkers in post-COVID-19 patients with reduced DLCO compared to those with normal DLCO suggests an additional impact of persistent endothelial dysfunction on gas exchange, beyond the reparative process of ARDS. Moreover, this potential impact is underscored by the association between ICAM-1 and ET-1 levels with patients' symptoms, including dyspnea, and quality of life six months post-ICU discharge.

Previous studies have shown that in COVID-19 related ARDS, there is a tendency toward a more severe phenotype related to extended pulmonary endothelial injury in the early phase of the disease [37]. Nevertheless, Ang-2 and ICAM-1 were the only markers observed to be elevated in non-survivors in contrast to survivors [38]. The swift and disproportionate rise in reaction to lung injury suggests an early disruption of inflammation and hemostasis, leading to further dysregulation later on [39]. Consistent with this, numerous biomarkers associated with the endothelial pathway in long COVID syndrome have been identified, including Ang-1, Ang-2, P-selectin, ICAM-1, and VEGF [38, 40–43]. In our study, we observed an increase in inflammatory proteins in patients with reduced gas transfer capacity. We speculate that the combination of these proteins may affect pulmonary vascular endothelium function.

A salient finding is the sustained elevation of systemic inflammation in our post-COVID-19 patients, evident through heightened C-reactive protein and inflammatory cytokine levels six months after ICU discharge. Notably, proinflammatory cytokines, known instigators of endothelial cell activation in COVID-19 patients [44], can lead to increased expression of adhesion molecules, IL-8, and CCL-2 [45]. Furthermore, inflammatory activation of endothelial cells triggers the NF-κB pathway, potentially associated with the production of inflammatory cytokines [46]. The release of chemoattractants by endothelial cells may enhance the recruitment of neutrophils and monocytes [47], as well as the extravasation of circulating cells [48], intensifying the inflammatory response.

The soluble fraction of CD40-L (sCD40-L) is known to play a crucial role in inflammation, particularly by upregulating the expression of cell adhesion molecules, proinflammatory cytokines, and chemokines [49]. The interaction of sCD40-L with endothelial cells, primarily through its canonical receptor CD40, influenced by TNF-α, modulates the release of reactive oxygen species, leading to an imbalance in nitric oxide levels, perpetuating endothelial dysfunction [50]. Notably, CD40-L is transiently expressed on T cells during inflammatory conditions, and its interaction with CD40 contributes to the initiation and progression of cellular and humoral adaptive immunity [51], forming a common pathway in the pathogenesis of autoimmune diseases [52, 53]. In our post-COVID-19 patients, T-cell activation is evident through increased expression of IFN-β which correlated CD69 on T cells, a widely used marker for activated T cells and a regulator of the immune response [54, 55]. Furthermore, CD69 expression is implicated in determining cytokine release patterns and the migration of activated lymphocytes [56, 57].

Furthermore, IFN-β assumes a pivotal role in robust antiviral defence and serves as a significant link between innate and adaptive immune responses [58, 59]. However, elevated levels of IFN-β can exert detrimental effects during viral infections. These effects may manifest through immunosuppression, hindering effective viral control [59], or by inducing inflammation and causing tissue damage that exacerbates the disease [60]. SARS-CoV-2 disrupts cellular signalling pathways, creating a conducive setting for viral replication that leads to a diminished IFN-β response, possibly attributed to inherent deficiencies within the type I IFN signalling cascade or the production of autoantibodies [61]. Conversely, our data demonstrates that post-COVID-19 patients with gas exchange impairment exhibit heightened T cell activity correlated with elevated levels of IFN-β in plasma. Several studies in post-COVID-19 patients have proposed diverse hypotheses to explain the persistent inflammatory state and elevated IFN-β levels [62], suggesting potential persistence of viral antigens driving immune stimulation [63].

Finally, the in vitro model used validates the impact of these plasma soluble proteins on endothelial function. Our data reveals a potential effect of sCD40-L and IFN-β on endothelial function by increasing the expression of ICAM-1 and caspase-1, a recognized indicator of pyroptosis [64, 65]. While no discernible differences in ET-1 are noted after plasma stimulation of healthy volunteers and post-COVID-19 patients, the reduction in ET-1 release upon IFN-β blockade underscores the pivotal role of this pathway. Furthermore, the expression of IFI16 mRNA, an interferon-inducible gene, signifies inflammatory activity in endothelial cells by mediating the expression of inflammasome components, ICAM-1, IL-8, and CCL-2 [66, 67].

Our study acknowledges several limitations. First, like many other studies, we were unable to assess the premorbid clinical status, making it impossible to rule out pre-existing impairment prior to admission. Second, direct access to endothelial samples from patients was not feasible due to the invasive nature of the procedure required for obtaining such samples. Third, samples from the same patients during the acute phase of ARDS secondary to COVID-19 were not available. Fourth, conventional endothelial reactivity tests were not employed due to their limitations in accurately representing the capillary vascular bed. Fifth, our in vitro model utilized a primary HUVECs cell line instead of endothelial cells isolated directly from patients.

## Conclusions

Our study reveals that 6 months after ICU discharge from severe ARDS secondary to COVID-19, patients with gas exchange impairment exhibit evidence of systemic inflammation, along with elevated ICAM-1 levels, ultimately resulting in increased plasma ET-1 levels. The persistent systemic inflammation observed in these patients may be attributed to increased activity of the NLRP3 inflammasome, leading to an increase in inflammatory cytokines, exacerbated by T-cell activation. Specifically, elevated levels of sCD40-L, along with persistent IFN-β-mediated viral inflammation, were observed. Both sCD40-L and IFN-β influenced endothelial function. Collectively, these findings provide insight into how systemic inflammation contributes to endothelial dysfunction, impairing gas exchange and perpetuating this alteration, impacting patients' symptomatology and quality of life months after acute infection.

### Supplementary Information

Additional file 1: Supplementary Tables. Table S1- [ELISA commercial references]. Table S2- [Primer sequences]. Table S3- [Clinical characteristics]. Table S4- [Current treatment]. Table S5- [Exercise capacity of ARDS survivors]; Supplementary Figures. Fig. S1- [Relation of ICAM-1 and ET-1 with DLCO parameters]. Fig. S2- [Inflammatory cytokines are released]. Fig. S3- [In vitro model of vascular endothelium]. Fig. S4- [In vitro model of vascular endothelium: mRNA expression]; Supplementary Methods.

## Data Availability

No personal identifier or personal data of patients will be shared following regulations related to patient’s privacy. De-identified clinical and laboratory findings data are available upon request to Carolina Cubillos Zapata (cubilloszapata@gmail.com).

## Abbreviations

ARDS: Acute respiratory distress syndrome
AUC: Area under the curve
BMI: Body mass index
CBA: Cytometric bead array
CCL-2: Chemokine C–C motif ligand 2
CD: Cluster differentiation
CD40-L: Cluster differentiation 40-ligand
CTRL: Control
DLCO: Diffusing capacity of the lungs for carbon monoxide
DPBS: Dulbecco’s PBS
ET-1: Endothelin-1
HUVECs: Human umbilical vein endothelial cells
HV: Healthy volunteers
ICAM-1: Intracellular adhesion molecule 1
ICU: Intensive care unit
IFI-16: Interferon-inducible protein 16
IFN- β: Interferon-β
IL-1β: Interleukin-1β
IL-6: Interleukin-6
IL-8: Interleukin-8
MFI: Mean fluorescence intensity
NF-κB: Nuclear factor- κB
NLRP3: Nucleotide-binding oligomerization domain receptor-like receptor 3
PBMCs: Peripheral blood mononuclear cells
PBS: Phosphate buffered saline
PCOV: Post-COVID-19 survivors
PLS-DA: Partial least squares-discriminant analysis
ROC: Receiver operating characteristics
SARS-CoV-2: Severe acute respiratory syndrome coronavirus-2
sCD40-L: Soluble fraction of CD40-L
SD: Standard deviation
SEM: Standard error of the mean
SF-12: Medical Outcomes Study 12-Item Short-Form Health Survey
TNF-α: Tumor necrosis factor-α
VA: Alveolar volume
VIP-scores: Variable importance projection scores
