# Supplementary material for: Endothelial dysfunction and persistent inflammation in severe post-COVID-19 patients: implications for gas exchange
Source: BMC Med. 2024 Jun 13;22:242. doi: 10.1186/s12916-024-03461-5 (PMC11170912; doi:10.1186/s12916-024-03461-5)
Supplement: Supplementary file 1 — Additional file 1: Supplementary Tables. Table S1- [ELISA commercial references]. Table S2- [Primer sequences]. Table S3- [Clinical characteristics]. Table S4- [Current treatment]. Table S5- [Exercise capacity of ARDS survivors]; Supplementary Figures. Fig. S1- [Relation of ICAM-1 and ET-1 with DLCO parameters]. Fig. S2- [Inflammatory cytokines are released]. Fig. S3- [In vitro model of vascular endothelium]. Fig. S4- [In vitro model of vascular endothelium: mRNA expression]; Supplementary Methods. [file 12916_2024_3461_MOESM1_ESM.docx]

**ADDITIONAL FILE 1**

**Title**: **Endothelial Dysfunction and Persistent Inflammation in Severe Post‑COVID-19 Patients: Implications for Gas Exchange**

**Authors**: Enrique Alfaro^1,2^, Elena Díaz-García^1,2^, Sara García-Tovar^1^, Raúl Galera^1,2^, Raquel Casitas^1,2^, María Torres-Vargas^1,2^, Cristina López-Fernández^1,2^, José M Añón^3^, Francisco García-Río^1,2,4,†^, Carolina Cubillos-Zapata^1,2,†^

^†^F.G-R. and C.C-Z. are joint supervisors of the study.

**Affiliations**:

^1^Respiratory Diseases Group, Respiratory Service, La Paz University Hospital, IdiPAZ, Madrid, Spain; ^2^Biomedical Research Networking Centre on Respiratory Diseases (CIBERES), Madrid, Spain; ^3^Department of Intensive Medicine, La Paz University Hospital, Madrid, Spain; and ^4^Faculty of Medicine, Autonomous University of Madrid, Madrid, Spain.

^†^**Correspondence**: Francisco García-Río, Paseo de la Castellana 261, Madrid, 28046, Spain; E-mail: fgr01m@gmail.com; and Carolina Cubillos-Zapata, Paseo de la Castellana 261, Madrid, 28046, Spain, E-mail: [cubilloszapata@gmail.com](mailto:cubilloszapata@gmail.com)

**SUPPLEMENTARY TABLES**

**Table S1**. Elisa commercial references

| ELISA target | Manufacturer | Reference | Detection limit |
| --- | --- | --- | --- |
| ICAM-1 | DIACLONE, France | 850.540.192 | 0.1 ng/mL |
| CCL-2 | DIACLONE, France | 873.030.096 | 5.8 pg/mL |
| ET-1 | Biotechne, USA | DET100 | 0.087 pg/mL |
| CD40L | Invitrogen, Austria | BMS293 | 0.06 ng/mL |
| IFN-β | CUSABIO technology, USA | CBS-E09889h | 31.25 pg/mL |
| CRP | Invitrogen, Austria | KHA0031 | 18.75 pg/mL |

**Table S2**. Primer sequences

| Target | Primer | Sequence |
| --- | --- | --- |
| ICAM-1 | Forward primer | CTCGTCCTCTGCGGTCAC |
|  | Reverse primer | TGAACCCCACAGTCACCTATG |
| IL-8 | Forward primer | ACTGAGAGTGATTGAGAGTGGAC |
|  | Reverse primer | AACCCTCTGCACCCAGTTTTC |
| IL-6 | Forward primer | GGTACATCCTCGACGGCATCT |
|  | Reverse primer | GTGCCTCTTTGCTGCTTTCAC |
| TNF-α | Forward primer | GGCGTGGAGCTGAGAGATAAC |
|  | Reverse primer | GGTGTGGGTGAGGAGCACAT |
| NLRP3 | Forward primer | TGCCCGTCTGGGTGAGA |
|  | Reverse primer | CCGGTGCTCCTTGATGAGA |
| caspase-1 | Forward primer | GGAAACAAAAGTCGGCAGAG |
|  | Reverse primer | ACGCTGTACCCCAGATTTTG |
| NF-κB | Forward primer | GGTGCGGCTCATGTTTACAG |
|  | Reverse primer | GATGGCGTCTGATACCACGG |
| IFI-16 | Forward primer | GAAGTGCCAGCGTAACTCCTA |
|  | Reverse primer | TACCTCAAACACCCCATTCAC |

**Table S3**. Clinical characteristics of the study subjects

| **Characteristic** | | **Overall ARDS survivors**  (n=88) | **ARDS survivors with normal DLCO**  (n=59) | **ARDS survivors with decreased DLCO**  (n=29) | **p-Value** |
| --- | --- | --- | --- | --- | --- |
| Ethnicity, n (%) | | | | | 0.323 |
|  | Caucasian | 44 (50.0) | 31 (52.5) | 13 (44.8) |  |
|  | Latin-American | 19 (21.6) | 13 (22.0) | 6 (20.7) |  |
|  | African American | 1 (1.1) | 0 | 1 (3.4) |  |
| Alcohol consumption (>10 g/day), n (%) | | 5 (5.7) | 3 (5.1) | 2 (6.9) | 0.583 |
| Smoking status, n (%) | | | | | 0.738 |
|  | Current | 1 (1.1) | 1 (1.6) | 0 |  |
|  | Former | 26 (29.5) | 18 (30.5) | 8 (29.6) |  |
|  | Never | 61 (69.3) | 40 (67.8) | 21 (72.4) |  |
| Current smoker before COVID, n (%) | | 11 (12.5) | 9 (15.2) | 2 (6.9) | 0.219 |
| ICU therapies, n (%) | | | | | |
|  | Systemic corticosteroids | 76 (86.4) | 52 (88.1) | 24 (82.8) | 0.485 |
|  | Vasopressor drugs | 86 (97.7) | 59 (100) | 27 (93.1) | 0.833 |
|  | Midazolam | 72 (81.8) | 52 (88.1) | 20 (69.0) | 0.575 |
|  | Propofol | 88 (100) | 61 (100) | 27 (100) | - |
|  | Dexmedetomidine | 81 (92.0) | 55 (93.2) | 26 (89.6) | 0.545 |
|  | Ketamine | 17 (19.3) | 13 (22.0) | 4 (13.8) | 0.537 |
|  | Fentanyl | 72 (81.8) | 51 (86.4) | 21 (72.4) | 0.138 |
|  | Remifentanil | 32 (36.4) | 22 (37.2) | 10 (34.4) | 0.223 |
|  | Cisatracurium | 80 (90.9) | 56 (94.9) | 24 (82.8) | 0.670 |
| Respiratory symptoms at 6 months from ICU discharge | | | | | |
|  | Respiratory symptoms, n (%) | 66 (75.0) | 42 (71.1) | 24 (82.8) | 0.045 |
|  | Dyspnea mMRC ≥ 2, n (%) | 36 (40.9) | 21 (35.6) | 15 (51.7) | 0.063 |
|  | Cough, n (%) | 25 (28.4) | 17 (28.8) | 8 (27.6) | 0.867 |
|  | Expectoration, n (%) | 17 (19.3) | 11 (18.6) | 6 (20.7) | 0.646 |
| Lung function at 6 months from ICU discharge | | | | | |
|  | FVC, l | 3.34 ± 0.93 | 3.37 ± 0.81 | 3.67 ± 1.11 | 0.189 |
|  | FVC, % pred. | 91 ± 16 | 90 ± 13 | 92 ± 20 | 0.658 |
|  | FVC, z-score | -0.66 ± 1.09 | -0.70 ± 0.94 | -0.60 ± 1.36 | 0.703 |
|  | FEV_1_, l | 2.86 ± 0.70 | 2.81 ± 0.66 | 2.95 ± 0.76 | 0.382 |
|  | FEV_1_, % pred. | 95 ± 15 | 96 ± 14 | 94 ± 17 | 0.657 |
|  | FEV_1_, z-score | -0.32 ± 1.00 | -0.28 ± 0.95 | -0.38 ± 1.12 | 0.656 |
|  | FEV_1_/FVC | 0.83 ± 0.06 | 0.84 ± 0.06 | 0.81 ± 0.07 | 0.070 |
|  | FEV_1_/FVC, z-score | 0.70 ± 0.87 | 0.80 ± 0.78 | 0.49 ± 1.01 | 0.126 |
|  | DLCO, mmol/min/kPa | 6.85 ± 1.78 | 7.16 ± 1.84 | 6.23 ± 1.50 | 0.021 |
|  | DLCO, % pred. | 87 ± 20 | 92 ± 20 | 77 ± 16 | <0.001 |
|  | DLCO, z-score | -0.92 ± 1.35 | -0.57 ± 1.31 | -1.63 ± 1.16 | <0.001 |
|  | DLCO/VA, mmol/min/kPa/l | 1.46 ± 0.30 | 1.53 ± 0.29 | 1.32 ± 0.29 | 0.002 |
|  | DLCO/VA, % pred. | 100 ± 19 | 104 ± 17 | 91 ± 20 | 0.002 |
|  | DLCO/VA, z-score | -0.04 ± 1.30 | 0.26 ± 1.16 | -0.64 ± 1.37 | 0.004 |
|  | VA, l | 4.77 ± 1.10 | 4.71 ± 1.03 | 4.88 ± 1.24 | 0.499 |
|  | VA, % pred. | 88 ± 18 | 89 ± 19 | 85 ± 15 | 0.427 |
|  | VA, z-score | -1.12 ± 1.41 | -1.04 ± 1.46 | -1.29 ± 1.31 | 0.434 |

Values are mean ± standard deviation or number (percentage) according to their type.

Abbreviations: ICU, intensive care unit; mMRC, modified Medical Research Council; FVC, forced vital capacity; FEV_1_, forced expiratory volume at 1 second; DLCO, diffusing capacity of the lung for carbon monoxide; VA, alveolar volume.

**Table S4**. Active Treatments Undertaken by Patients at the Time of Study

| **Current treatment** | | **Overall ARDS survivors**  (n=88) | **ARDS survivors with normal DLCO**  (n=59) | **ARDS survivors with decreased DLCO**  (n=29) | **p-Value** |
| --- | --- | --- | --- | --- | --- |
|  | Heparin, n (%) | 3 (3.4) | 1 (1.7) | 2 (6.9) | 0.252 |
|  | Direct oral anticoagulants, n (%) | 23 (26.1) | 16 (27.1) | 7 (24.1) | 0.489 |
|  | Antiaggregant drugs, n (%) | 5 (5.7) | 3 (5.1) | 2 (6.9) | 0.535 |
|  | Long-term oxygen therapy, n (%) | 4 (4.5) | 1 (1.7) | 3 (10.3) | 0.103 |
|  | Systemic corticosteroids, n (%) | 4 (4.5) | 2 (3.4) | 2 (6.9) | 0.401 |
|  | Inhaled corticosteroids, n (%) | 13 (14.8) | 8 (13.6) | 5 (17.2) | 0.435 |
|  | Long-acting muscarinic antagonist, n (%) | 6 (6.8) | 2 (3.4) | 4 (13.8) | 0.104 |
|  | Long-acting beta-2 agonists, n (%) | 15 (17.0) | 8 (13.6) | 7 (24.1) | 0.173 |
|  | N-acetylcysteine, n (%) | 2 (2.3) | 2 (3.4) | 0 | 0.447 |
|  | Antihistamine drugs, n (%) | 13 (14.8) | 11 (18.6) | 2 (6.9) | 0.125 |
|  | Angiotensin-converting enzyme inhibitors, n (%) | 28 (31.8) | 17 (28.8) | 11 (37.9) | 0.266 |
|  | Calcium channel antagonists, n (%) | 15 (17.0) | 12 (20.3) | 3 (10.3) | 0.194 |
|  | Beta blockers, n (%) | 13 (14.8) | 8 (13.6) | 5 (17.2) | 0.435 |
|  | Diuretic drugs, n (%) | 13 (14.8) | 9 (15.3) | 4 (13.8) | 0.565 |
|  | Aldosterone antagonists, n (%) | 1 (1.1) | 0 | 1 (3.4) | 0.330 |
|  | Angiotensin II receptor antagonists, n (%) | 14 (15.9) | 12 (20.3) | 2 (6.9) | 0.091 |
|  | Antidiabetic drugs, n (%) | 14 (15.9) | 10 (16.9) | 4 (13.8) | 0.482 |
|  | Insulin, n (%) | 1 (1.1) | 1 (1.7) | 0 | 0.670 |
|  | Statins, n (%) | 37 (42.0) | 25 (42.4) | 12 (41.4) | 0.558 |
|  | Paracetamol, n (%) | 42 (47.7) | 29 (49.2) | 13 (44.8) | 0.439 |
|  | Metamizole, n (%) | 16 (18.2) | 11 (18.6) | 5 (17.2) | 0.561 |
|  | Non-steroidal anti-inflammatories, n (%) | 5 (5.7) | 5 (8.5) | 0 | 0.128 |
|  | Benzodiazepines, n (%) | 12 (13.6) | 10 (16.9) | 2 (6.9) | 0.169 |
|  | Opioids, n (%) | 5 (5.7) | 4 (6.8) | 1 (3.4) | 0.465 |
|  | GABA analogs, n (%) | 8 (9.1) | 6 (10.2) | 2 (6.9) | 0.473 |
|  | Antidepressants, n (%) | 4 (4.5) | 3 (5.1) | 1 (3.4) | 0.599 |
|  | Thyroid hormones, n (%) | 15 (17.0) | 10 (16.9) | 5 (17.2) | 0.596 |
|  | Proton pump inhibitors, n (%) | 32 (36.4) | 26 (44.1) | 6 (20.7) | 0.026 |
|  | Calcium and vitamin D, n (%) | 11 (12.5) | 10 (16.9) | 1 (3.4) | 0.066 |

Values are number (percentage).

Abbreviations: GABA, gamma-Aminobutyric acid.

**Table S5**. Comparison of the exercise capacity of ARDS survivors according to their DLCO at 6 months after ICU discharge

|  | **Subjects with decreased DLCO**  (n=29) | **Subjects with normal DLCO**  (n=59) | **p** |
| --- | --- | --- | --- |
| W peak, % pred. | 60 ± 22 | 69 ± 19 | 0.028 |
| BR peak, % | 45 ± 15 | 46 ± 12 | 0.828 |
| BF peak, min-1 | 34 ± 9 | 35 ± 8 | 0.657 |
| ∆VE/∆VCO2 peak | 36.5 ± 6.6 | 32.6 ± 4.5 | 0.003 |
| VD/VT peak, % | 25.7 ± 6.7 | 22.0 ± 6.2 | 0.018 |
| SaO2 peak, % | 95 ± 4 | 97 ± 5 | 0.007 |
| HRR, min-1 | 31 ± 17 | 31 ± 17 | 0.777 |
| VO2 peak, % pred. | 57 ± 14 | 65 ± 16 | 0.040 |
| RER peak | 1.18 ± 0.10 | 1.16 ± 0.11 | 0.643 |
| AT, % VO2max | 66 ± 14 | 68 ± 15 | 0.509 |
| ∆ EELV, l | -0.71 ± 0.79 | -0.63 ± 0.84 | 0.517 |
| ∆Borg/∆VO2 slope | 0.691 ± 0.346 | 0.479 ± 0.280 | 0.007 |
| ∆Borg/∆VO2 threshold | 4.1 ± 1.3 | 4.4 ± 5.4 | 0.578 |
| Maximal load of dyspnea | 5.8 ± 1.9 | 5.0 ± 2.6 | 0.150 |
| ∆VE/∆VCO2 peak > 35 | 15 (55.6) | 18 (26.9) | 0.009 |
| VD/VT peak > 25 | 14 (51.9) | 15 (22.7) | 0.007 |

Values are mean ± standard deviation or number (percentage) according to their type.

Abbreviations: W, work intensity; BR, breathing reserve; BF, respiratory frequency; ∆VE/∆VCO_2_, ventilatory equivalent for carbon dioxide; V_D_/V_T_, ratio of physiologic dead space to tidal volume; SaO_2_, oxygen saturation; HRR, heart rate reserve; VO_2_, oxygen uptake; RER, respiratory exchange ratio; AT, anaerobic threshold; ∆EELV, change from baseline in end-expiratory lung volume; ∆Borg/∆VO2, relationship between Borg scale dyspnea score and oxygen uptake.

**SUPPLEMENTARY FIGURES**

**Figure S1. Relation of ICAM-1 and ET-1 with DLCO parameters.** a) Spearman correlation of ICAM-1 plasma concentration from post-COVID-19 patients and DLCO % pred (upper panel) and DLCO/VA ratio (lower panel). b) Spearman correlation of ET-1 concentration in plasma from post-COVID-19 patients and DLCO % pred (upper panel) and DLCO/VA ratio (lower panel). Spearman correlation coefficients (ρ) and p-values (p) are indicated.

**Figure S2.** **Inflammatory cytokines are released by monocytes from post-COVID-19 patients with impaired gas exchange**

Enriched monocytes from post-COVID-19 patients with normal (n=45) and low (n=23) DLCO were cultured during 16 hours. The supernatants from these cell cultures were collected. a) IL-1β and (b) TNF-α production were quantified using a cytometric bead array analysis by flow cytometry. Violin plot: median ± quartiles. Mean differences were analyzed by Mann-Whitney U test.

**Figure S3. *In vitro* model of vascular endothelium.** HUVECs were cultured for 24 hours in presence of IFN-β (1ng/mL) or CD40-L (4 ng/mL) (n=6). a) Percentage of HUVECs with high expression of membrane ICAM-1 measured by flow cytometry. b) Percentage of HUVECs positive for active caspase-1 measured by flow cytometry. c) Endothelin-1 concentration in supernatant cell culture. Bars represent mean ± standard error of the mean (SEM). Differences of mean are analyzed by one-way ANOVA and multiple comparisons by Tukey’s test. p value is represented: *p<0.05, **p<0.01 against untreated HUVECs.

**Figure S4. *In vitro* model of vascular endothelium: mRNA expression**

HUVECs were cultured for 24 hours in presence of 10% plasma from healthy volunteers (HV, green, n=4) or post-COVID-19 patients (PCOV, orange, n=5). Plasmas were added untreated (CTRL) or previously treated for 16h with anti-IFN-β antibody (α-IFN-β, 8 μg/mL), anti-CD40-L antibody (Toralizumab, 8 μg/mL) or both. The relative mRNA expression of ICAM-1 (a), IL-8 (b), IL-6 (c), NLRP3 (d), caspase-1 (e), NF-κB (f) and IFI-16 (g) were analyzed by quantitative PCR. Bars represent mean ± standard error of the mean (SEM). Differences of mean are analyzed by two-way ANOVA and multiple comparisons by Sidak’s test. p value is represented: * p<0.05, ** p<0.01, *** p<0.001.

**SUPPLEMENTARY METHODS**

**Study subjects**

We recruited 88 consecutive patients aged 18 years or older who had survived COVID-19­-associated severe ARDS (defined according to the Berlin criteria)[14], requiring invasive mechanical ventilation for at least 7 days, with SARS-CoV-2 infection confirmed at that time by positive reverse-transcriptase polymerase chain reaction on nasal swab or tracheal aspirate. Exclusion criteria were persistent tracheostomy, need for permanent noninvasive ventilatory support, moderate-severe psychiatric conditions or cognitive deficits, hemodynamic instability, severe myopathy preventing ambulation, history of pulmonary resection, or respiratory infection in the 4 weeks prior to study testing.

All participants provided their written consent, and the study was approved by the institutional Ethics Committee (PI-4189).

**Data collection about severity of illness and course in the ICU**

Data regarding demographic variables, PaO2/FiO2 ratio and APACHE II score[15] at the time of ICU admission, mechanical ventilation parameters (plateau pressure, positive expiratory pressure, positive end-expiratory pressure, driving pressure, static compliance, and resistance), prone cycles required and the need for extracorporeal membrane oxygenation (ECMO) support, tracheostomy or reintubation were retrospectively registered from the patient’s electronic medical record. The treatment drugs (hypnotics, analgesics, muscle relaxants, corticoids and vasopressors), the complications developed (pressure ulcers, nosocomial infection, pleural effusion, deep venous thrombosis, pulmonary embolism, ischemic stroke, hyperactive delirium or ICU-acquired weakness), and ICU readmission were also recorded as well as the time of mechanical ventilation, duration of ICU stay, and duration of hospitalization.

**Clinical and functional assessment at 6 months after ICU discharge**

Anthropometric characteristics were measured, including body composition (BF511 monitor, Omron Healthcare, Kyoto, Japan). Based on self-administered questionnaires and medical records, smoking status and comorbidities were recorded. Current smokers were defined as subjects who currently smoked more than 10 cigarettes per day. Past smokers who had quit smoking were not considered smokers in this study. All medications used by the participants at the time of the clinical evaluation were also listed.

The respective questions of the European Community for Coal and Steel (ECCS) Questionnaire were used to identify respiratory symptoms (cough, chronic expectoration, dyspnea, wheezing or chest tightness)[16]. The degree of dyspnea was evaluated by the modified Medical Research Council (mMRC) dyspnea scale which ranges between 0 and 4[17].

Health-related quality of life was evaluated with the Spanish version of the Medical Outcomes Study 12-Item Short-Form Health Survey (SF-12), which includes eight multi-item domains that assess physical functioning (PF), social functioning (SF), role limitation due to physical problems (RP), role limitation due to emotional problems (RE), mental health (MH), bodily pain (BP), vitality (VT) and general health (GH). These domains are summarized in a physical component scale and a mental component scale. Scores for each aspect can range from 0 (worst) to 100 (best), and are standardized to a mean score of 50 in the general population, so that a score of less than 50 reflects worse than average HRQoL[18].

Spirometry and measurement of diffusing capacity of the lungs for carbon monoxide (DLCO) were performed using a MasterScreen PFT system (Viasys, CareFusion, Würzbourg, Germany) equipped with the SentrySuiteTM software, according to current standardization[19, 20]. Global Lung Initiative (GLI) equations were used as reference values[21, 22] and tests were interpreted according ERS/ATS technical strategies[23].

A symptom-limited incremental cycle exercise test was conducted on an electronically braked cycle ergometer (Vintus CPX, Carefusion) following standards of the ATS/ACCP statement[24], as previously described[25, 26]. Equipment was calibrated immediately before each test. The initial 2 min consisted of resting data collection followed by 1 min of unloaded cycling. Subsequently, workload was increased by 15 W/min until maximal symptom-limited exercise was achieved. Pedaling rates were maintained between 50 and 60 revolutions per minute. Expired gases and ventilation were measured on a metabolic cart that uses a pneumotachograph positioned at the mouth with O_2_ and CO_2_ analyzers. This allowed for breath-by-breath measurements of oxygen uptake (VO_2_), carbon dioxide production (VCO_2_), minute ventilation (V_E_), respiratory rate (BF), and tidal volume (V_T_). In all subjects, heart rate, heart rhythm, blood pressure, and oxygen saturation were also continuously monitored. Peak work rate (Wpeak) was defined as the highest work rate that the subject was able to maintain for at least 30 s. Anaerobic threshold (AT) was estimated using the nadirs of ventilatory equivalents and the V-slope method; both methods were used concurrently looking for consistency[24]. If AT was clearly discernible using either of the noninvasive methods, this value was reported. When differences in AT were observed between both techniques, the average value was used. However, in situations in which AT was not discernible using either method, the AT was categorized as indeterminate. The predicted values of Puente-Maestu and coworkers were used for the exercise measurements[27].

Changes in operational lung volumes were evaluated from duplicate measurements of inspiratory capacity (IC) at rest and every 2 min during exercise[28]. If efforts appeared submaximal or if anticipatory changes in breathing pattern occurred immediately preceding a maneuver, then the IC was not accepted. EELV was calculated as total lung capacity (TLC) minus IC. To minimize the variability of isolated EELV measurements, we considered that the patient had developed DH when the slope of linear regression of the EELV as a function of time was higher than zero[29]. In addition, the difference between the last EELV measurement, near end-exercise, and the resting value was computed.

By pointing to the Borg scale, subjects rated dyspnea at rest, every two minutes during exercise, and at peak exercise. To standardize for stimulus intensity, the Borg dyspnea scores were related to oxygen uptake. The exercise response slopes, expressed as means of slopes from linear regression analysis of individual subjects’ data, were used as indices of exertional dyspnea. Dyspnea “thresholds” were expressed as the x-intercepts of the relationships between Borg ratings and VO2[25].

**Flow Cytometry**

PBMCs were treated using the Transcription Factor Buffer Set (Becton-Dickinson Biosciences, NJ, USA) following manufacturer’s protocol. Cells were labeled for 30 minutes at 4ºC with antibodies against CD14 (Ref: 555399; Becton-Dickinson Biosciences, RRID:AB_398596), CD4 (Ref: 4A-100T; Immunostep, Salamanca, Spain), CD69 (Ref: 555531; Becton-Dickinson Biosciences, RRID:AB_395916) and NLRP3 (Ref:130-111-209; Miltenyi Biotec, Bergisch Gladbach, Germany, RRID:AB_2653035). Cells were acquired using BD FACS-Celesta flow cytometer (Becton-Dickinson Biosciences).

HUVECs were harvested from culture using EDTA 1mM for 30 minutes at 4ºC to avoid interference with adherence molecules in cell surface. Cells were washed in PBS and stained with antibody against ICAM-1 (Ref: 564685, Becton-Dickinson Biosciences, AB_273889) for 30 minutes at 4ºC and acquired using BD FACS-Celesta flow cytometer. To stain active caspase-1 in HUVECs, FAM-FLICA Caspase-1 assay kit (Ref: ICT098, Bio-Rad Laboratories, CA, USA) was used following manufacturer’s instructions. FAM-FLICA Caspase-1 assay employs fluorescent inhibitor probe FAM-YVAD-FMK to specifically label active Caspase-1 in living cells. HUVECs stained with FAM-FLICA were acquired using BD FACS-CALIBUR flow cytometer (Becton-Dickinson Biosciences, RRID:SCR_000401). All cytometry data were analyzed using FlowJo vX.0.7 software (Becton-Dickinson Biosciences).

**mRNA isolation and qPCR analysis**

RNA was extracted using High Pure RNA Isolation Kit (Roche Diagnostics, Basel, Switzerland). 0.25 μg of RNA was retro-transcribed using High-Capacity cDNA Reverse Transcription kit (Applied Biosystems, MA, USA). Gene expression was quantified by RTqPCR using NZY Supreme qPCR Green MasterMix (Nzytech, Lisboa, Portugal) and specific primers for targeted genes synthesized by Eurofins Genomics Srl (Vimidrone, Italy) (Supplementary table SII). RTqPCR was performed using CFX96 Touch Real-Time PCR Detection System (Bio-Rad Laboratories, RRID:SCR_018064) and results normalized to housekeeping gene 18S expression.
